# Pathophysiology, Diagnosis, and Treatment of Patients with Concomitant Severe Aortic Stenosis and Coronary Artery Disease: A Closer Look to the Unresolved Perplexity

**DOI:** 10.3390/jcm10081617

**Published:** 2021-04-11

**Authors:** Giuseppe Di Gioia, Jozef Bartunek, Tullio Tesorio, Vladan Vukcevic, Srdjan Aleksandric, Milan Dobric, Danilo Franco, Emanuele Barbato, Marko Banovic

**Affiliations:** 1 Division of Cardiology, Department of Advanced Biomedical Sciences, Federico II University, 80131 Naples, Italy; francodnl88@gmail.com (D.F.); emanuele.barbato@unina.it (E.B.); 2 Montevergine Clinic, 83013 Mercogliano, Italy; tulliotesorio@gmail.com; 3 Cardiovascular Center Aalst, OLV Clinic, 9300 Aalst, Belgium; jozef.Bartunek@olvz-aalst.be; 4 Belgrade Medical School, University of Belgrade, 11000 Beograd, Serbia; vladan.vukcevic@gmail.com (V.V.); srdjanaleksandric@gmail.com (S.A.); iatros007@gmail.com (M.D.); 5 Cardiology Department, University Clinical Center of Serbia, 11000 Beograd, Serbia

**Keywords:** aortic stenosis, coronary artery disease, TAVI

## Abstract

Degenerative aortic stenosis (AS) and coronary artery disease (CAD) are the most prevalent cardiovascular diseases in developed countries, and they coexist in up to 50% of patients. The pathophysiological rationale behind concomitant AS and CAD is discussed in detail in this review, together with prognostic implications. Detecting CAD in patients with AS may be challenging, as AS may mask the existence and symptoms of CAD. The safety and reliability of invasive and non-invasive physiological assessment for epicardial coronary disease are also a matter of debate. Finally, the selection and timing of optimal treatment of CAD in patients with severe AS are still unclear. Given the aging of the population, the increase in the prevalence of AS, and the ongoing paradigm shift in its treatment, controversies in the diagnosis and treatment of CAD in the setting of AS are deemed to grow in importance. In this paper, we present contemporary issues in the diagnosis and management of CAD in patients with severe AS who are transcatheter aortic valve implantation (TAVI) candidates and provide perspective on the treatment approach.

## 1. Introduction

Degenerative aortic stenosis (AS) and coronary artery disease (CAD) are among the most prevalent cardiovascular diseases in industrialized countries, and their co-occurrence is common [1,2]. Detecting CAD in patients with hemodynamically significant asymptomatic AS may be challenging, as AS may mask the existence and symptoms of CAD. Given the expected aging of the population, the increase in the prevalence of AS, and the ongoing paradigm shift in its treatment, controversies in the diagnosis and treatment of CAD in the setting of AS will grow in importance [3,4,5]. The concerns encompass the diagnosis of CAD, the assessment of the severity of epicardial artery stenosis, and the selection and timing of optimal treatment of CAD. The latter is particularly important as, unlike patients undergoing surgical aortic valve replacement (SAVR), the most appropriate management of CAD in patients undergoing percutaneous transcatheter aortic valve implantation (TAVI) is yet to be codified. In fact, the impact of CAD and its treatment with percutaneous coronary intervention (PCI) on clinical outcome remains unclear in these patients [6,7,8].

In this paper, we present contemporary issues in the diagnosis and management of CAD in patients with severe AS who are TAVI candidates, and provide perspective on the treatment approach.

## 2. Pathophysiological Rationale behind AS and CAD Co-Existence

The pathophysiological basis behind both degenerative AS and epicardial CAD is atherosclerosis. The connection is so penetrative that the calcification of the aortic valve has been proposed as a surrogate marker of CAD [9]. This also implies that similar cardiovascular risk-factors are involved in both diseases; including age, male gender, arterial hypertension, increased lipid levels, diabetes and chronic kidney disease [10,11]. Additional parameters, such as unfavorable genetic predisposition, also play a significant role. Since both diseases share common risk factors it is not surprising that up to 50% of patients have concomitant AS and CAD, a percentage that rises to as high as 75% if only patients who are planned for TAVI are considered [8,12,13]. According to the Society of Thoracic Surgeons (STS)/American College of Cardiology (ACC) Transcatheter Valve Therapy (TVT) registry, 63% of patients undergoing TAVI in the USA have significant CAD, of which approximately 1/3 have multivessel disease [14]. Surprisingly, in recently published randomized trials comparing TAVI vs. SAVR in low-risk symptomatic AS patients, CAD is poorly characterized. Actually, in the Partner 3 trial [15] there are no data regarding prior CABG or PCI in included patients, whereas in the Evolut low-risk trial [16], the prevalence of CAD in enrolled patients is not reported. Furthermore, in the latter trial significant CAD (Syntax score > 22) was an exclusion criterion.

The primum movens in the pathogenesis of degenerative AS is endothelial damage that allows the infiltration of lipids, specifically low-density lipoproteins (LDL) and lipoprotein (a) into the fibrosa and triggers the accumulation of inflammatory cells into the aortic valve [17]. Macrophages, mast cells, CD4+ T cells and CD8+ T cells have been identified in surgically removed calcific aortic valves [18]. The endothelial injury may be caused by several factors, predominantly by blood flow-related mechanically induced stress, but also due to different cytokines and lipid-derived species. The uncoupling of nitric oxide synthase (NOS) induces the release of reactive oxygen species (ROS), which promotes the oxidation of lipids and the secretion of cytokines. Due to the increased production of metalloproteinases and lower synthesis of tissue inhibitors of these enzymes, disorganized fibrous tissue accumulates within the aortic valve. Microcalcifications develop early in the course of the disease, mainly driven by the microvesicles secreted by valvular interstitial cells (VIC) and macrophages [17,19]. Pronounced expression of different enzymes and proteins (including ecto-nucleotidases NPP1; 5¨-NT, ALP; and bone morphogenetic protein 2) may be responsible for osteogenetic transdifferentiation. Further process of aortic valve calcification is mainly coordinated by Osteoblast-like cells. In addition, the deposition of mineralized matrix is accompanied by fibrosis, neovascularization and further recruitment of inflammatory cells [17,19]. Finally, pathological studies have confirmed that several types of foam cells, which represent the hallmark of early atherosclerotic process, may be found in the endothelium of both the epicardial coronary arteries and the aortic valve leaflets as early as the second and third decades of life [10,20]. Apparently, the pathophysiological mechanisms of AS and CAD share many similarities, especially in the beginning. However, the clinical onset of CAD might be unstable and sudden and is often triggered by non-obstructive plaque rupture, whereas the process of aortic valve calcification/immobilization is gradual and stable, up to the advanced stages of the disease.

## 3. Detecting CAD in the Setting of AS

In symptomatic AS chest pain may be caused by both CAD and AS. However, symptoms of angina pectoris have a low positive predictive value for CAD in patients with AS, as <50% of patients with AS and typical angina have significant epicardial coronary artery lesions [1]. In those without obstructive CAD, angina may be the result of mismatch between the need and the actual supply of oxygen, due to its increased demand caused by elevated chronic afterload and subsequent increased wall stress, wall thickening, structural changes in coronary microcirculation, and the impairment in coronary flow reserve [21,22,23,24]. In symptomatic AS patients exercise testing is contraindicated. On the other hand, in asymptomatic patients, stress-echocardiography and cardiopulmonary exercise testing may unmask symptoms and latent CAD. Albeit stress testing has been proven to be safe in asymptomatic patients, some resistance is still observed among cardiologists, as shown by the fact that in the recently published Valvular Heart Disease Survey II, it was performed only in a total of 25 patients (6.1%) out of 409 patients with AS and NYHA class I [25].

The negative predictive value/specificity of exercise-induced angina is also suboptimal (70–80%), with false positive results related to myocardial hypertrophy [26]. Finally, significant CAD might be present without angina even in patients with severe AS [26,27,28]. Exercise-induced ECG changes are often unreliable due to already existing repolarization abnormalities [27]. Low-dose (up to 20 µg/mL/min) dobutamine stress-echocardiography might also be useful for additional regional LV kinetics analysis in low-flow AS [29]. Dipyridamole stress-echocardiography is a safe and feasible tool in patients with severe AS to exclude significant CAD, but a positive test has relatively low value in predicting CAD [30]. Nevertheless, whatever type of stress-test is used, if it is positive for ischemia the patient should be referred to coronary angiography prior to AVR.

One out of five patients with severe AS and normal exercise testing may have latent significant obstructive CAD [31]. This may support a pro-active use of non-invasive imaging such as multi-slice computed tomography (MDCT) coronary angiography as a screening tool. This would be particularly convenient since MDCT has been regularly performed preoperatively to evaluate vascular access, annular and aortic root measurements, and anatomical features in patients undergoing TAVI. Interestingly, Chieffo and colleagues reported that only 22% of patients required additional coronary angiography after first-line CAD screening with MDCT in a single center experience [32]. Plasma B-type natriuretic peptide levels may also be a complementary tool to exercise testing for risk stratification of patients with AS and/or left ventricular hypertrophy [33,34,35]. BNP values >118 pg/mL showed sensitivity/specificity of 63% and 73%, respectively, in unmasking CAD in AS patients [31]. In fact, ischemia may cause increased ventricular volume and wall stress, leading to elevations in BNP. It is also possible that elevations in BNP reflect increased ventricular filling pressures and non-compliant LV, which are particularly expressed in severe AS patients. However, in current ESC guidelines for the treatment of valve diseases, markedly elevated resting BNP has been introduced as an indication for AVR in asymptomatic AS patients (IIa C; 1) [33].

Given the limitations of non-invasive techniques, coronary angiography is often the method of choice for diagnosing CAD in AS patients. It is mostly used in candidates for aortic valve replacement (AVR) usually in men older than 40 and women older than 50 years. With the current predominance of degenerative AS and increased age of patients undergoing AVR, coronary angiography should therefore be considered in the vast majority of patients with degenerative AS.

## 4. Assessment of Functional Significance of Epicardial Coronary Artery Lesion in Patients with Significant Aortic Stenosis

**Figure 1:**
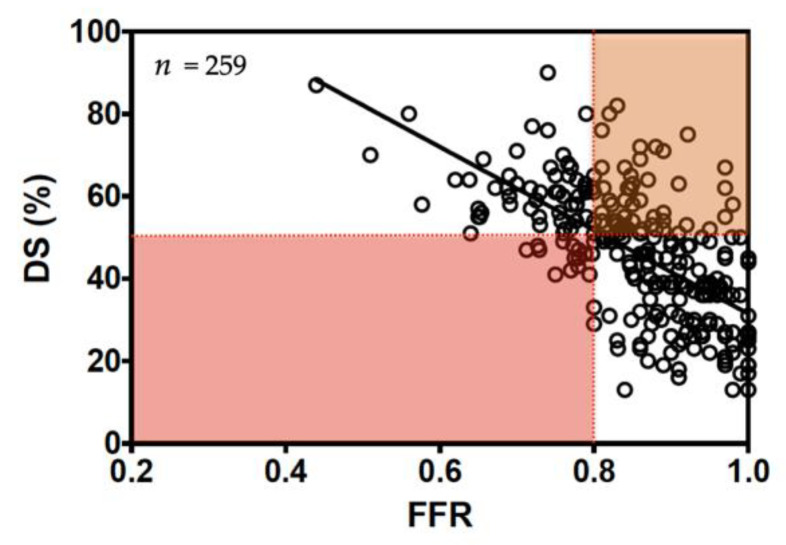
Correlation between diameter stenosis (DS) and fractional flow-reserve (FFR) in patients with aortic stenosis and stable coronary artery disease (CAD) (modified from Di Gioia et al. Am. J. Cardiol. 2017; 120(1):106–110).

The use of intracoronary physiological assessment to detect ischemia-producing coronary stenosis in the setting of severe AS is still a matter of debate. On the one hand, coronary physiology guidance has consistently been associated with good clinical outcomes in patients with stable CAD [36,37,38]. On the other hand, structural abnormalities normally present in patients with severe AS may hamper the reliability of these techniques. As a matter of fact, despite CAD being present in around 50% of patients with severe AS, angina is commonly observed also in patients without epicardial coronary obstructions, and is associated with increased risk of sudden death [39]. The reduced coronary flow reserve (CFR) in these patients is thought to be caused by left ventricular hypertrophy (LVH), elevated LV-cavity pressures, and reduced diastolic perfusion time. Interestingly, differently from hypertrophic cardiomyopathy, microvascular impairment seems to be related to external compression forces rather than small vessels disease [23,40,41]. In fact, CFR improves acutely after TAVI or surgical AVR, whereas the remaining hypertrophy continues to exert a limited influence on physiology [42]. This is consistent with the evidence that subendocardial ischemia on PET scanning is proportional to the aortic valve pressure gradient rather than the extent of left ventricular hypertrophy [23]. The impaired hyperemic response to adenosine, and the necessity of vasodilators administration has shed some doubts on the safety and reliability of fractional flow reserve (FFR) measurements in AS patients. However, the identification of ischemia-producing stenosis remains an important clinical need, also in the TAVI era. In fact, in patients with AS, non-invasive functional evaluation is often impractical or inconclusive and the decision-making process on coronary revascularization is often based on angiographic evaluation alone [43]. Yet, in AS patients the correlation between invasive functional and angiographic evaluation is moderate at best, and the assessment of CAD severity based on angiography poorly predicts the hemodynamic significance (Figure 1) [43]. Revascularization with concomitant CABG surgery and SAVR reduces the risks of perioperative myocardial infarction as well as long-term morbidity and mortality in patients with concomitant CAD [31,44]. Thus, it is particularly important to define which patients to revascularize in the TAVI era.

As previously discussed, the issues against FFR measurements in AS can be summarized as follows:

(1) There are doubts regarding the safety of vasodilators administration in patients with severe AS;
(2) Coronary flow reserve is impaired in these patients, with reduced hyperemic response;
(3) CFR impairment reverts virtually instantaneously after TAVI or SAVR.

For these reasons, it has been suggested that using non-hyperemic pressure ratios (NHPR), such as instantaneous wave-free ratio (iFR) may be convenient in patients with severe AS, as they do not require either vasodilator administration or hyperemia [45]. In AS, though, microvascular resistances are lower at rest as compared to patients without AS, contributing, in part, to the impaired CFR. In turn, this may lead to overestimation of lesion severity by iFR [46,47]. In a study by Ahmad et al. conducted in 28 patients (30 lesions), flow during the wave-free period of diastole did not change post-TAVI, whereas whole-cycle hyperemic flow increased significantly [48]. This translated in stable IFR values and significantly reduced FFR values after TAVI, suggesting that FFR might underestimate lesion severity in these patients. On the contrary, Pesarini et al. showed no significant overall change in FFR before and after TAVI (0.89 ± 0.10 versus 0.89 ± 0.13; *p* = 0.73), with only 8 out of 133 assessed lesions (6%) crossing the threshold of 0.8. Interestingly, a different trend was found for FFR positive and negative lesions after TAVI. The former seemed to worsen (0.71 ± 0.11 versus 0.66 ± 0.14), whereas negative FFR values improved after TAVI (0.92 ± 0.06 versus 0.93 ± 0.07) [49]. These results were maintained when FFR was measured at least 6 months after valve replacement, and if confirmed in larger studies they would allow the exclusion of ischemia in AS patients with clearly normal FFR (>0.85) [50]. The results of these studies are in line with the evidence that the difference between resting distal coronary vs. aortic pressure ratio (Pd/Pa) and FFR (Pd/Pa–FFR) seems not to be influenced by aortic valve area, gradient or LVH, but rather by the presence of diabetes, peripheral vascular disease or chronic kidney disease [51].

The same group evaluated acute variations of iFR after TAVI in 145 lesions (66 patients). Mean IFR did not change after TAVI, though individual IFR values varied widely resulting in 15% of lesions crossing the 0.89 threshold. The diagnostic accuracy of IFR in predicting FFR <0.8 was only 65% [52].

Different iFR cut-offs have been proposed in patients with AS. In a study by Yamanaka et al. [53], an iFR value of 0.82 was found to be the best predictor of ischemia with regards to both iFR and perfusion scintigraphy. In another study [54], FFR correlated with scintigraphy significantly better than IFR (agreement 85% vs. 59%, respectively; *p* = 0.014), whereas an iFR threshold of 0.82 increased the agreement with scintigraphy to 73%.

Clinical data on physiology-guided revascularization in patients with AS are scarce. In line with what has been shown in other clinical settings [55], in a propensity matched study, the use of FFR in patients with moderate or severe AS and at least one intermediate coronary lesion significantly impacted clinical practice, resulting into deferral of aortic valve replacement, more patients treated with PCI, and in patients treated with CABG, into less venous grafts and anastomoses without increasing adverse events up to five years [56]. In another retrospective study conducted in patients undergoing TAVI, FFR-guidance was associated with better major adverse cardiovascular and cerebrovascular event-free survival compared with the angio-guided group at two years (92.6% vs. 82%; *p* = 0.035) [57].

In order to overcome the necessity to administer vasodilator, a hybrid iFR-FFR approach has been proposed and evaluated. A “defer iFR value” of >0.93 yielded a negative predictive value of 98.4% to exclude FFR non-significant stenoses (>0.8), whereas a “treatment iFR value” <0.83 had a positive predictive value of 91.3%. This approach allowed 63% of patients to be spared from adenosine, whilst maintaining 97% overall agreement with FFR [58].

In conclusion, the body of evidence regarding the use of physiology to assess coronary stenoses in patients with AS is rapidly growing, but at the moment it consists mainly of observational and retrospective studies. Future randomized studies will further clarify when and how to use physiology in this setting. It is therefore important to always keep in mind the clinical presentation of the patient, including the type and intensity of symptoms, the location of coronary stenosis (e.g., left main, proximal LAD), and the expected clinical benefit before even considering physiological assessment.

## 5. Treatment of CAD in Patients with AS

The comparative efficacy of SAVR vs. TAVI has been thoroughly evaluated in several large randomized trials and match-based observational studies [15,16,59,60,61]. Yet, in the vast majority of trials, patients with CAD requiring revascularization were either excluded, or included in small numbers without thorough analysis, creating a gap in evidence [15,16]. For example, in the Partner 3 trial concomitant coronary revascularization was performed in 6.5% and 12.8% of patients, respectively [15]. According to current European guidelines, patients with severe AS associated with significant CAD (stenosis > 50 to 70% of vessel luminal diameter) undergoing SAVR should be treated by combined SAVR and coronary artery bypass grafting (CABG), as CABG at the same time of SAVR decreases mortality in comparison to SAVR alone [33,62]. Also, SAVR after CABG is associated with significantly increased risk so common sense and consensus dictate the treatment of both diseases simultaneously to avoid repeated sternotomy [63]. For the same reason, patients with moderate AS planned for CABG should also undergo SAVR during the same procedure. So far, there are limited data regarding outcomes of TAVI/PCI vs. SAVR/CABG. For patients with severe CAD, especially for those with affected LAD, the SAVR + LIMA-LAD option has set a high bar. Nevertheless TAVI + PCI in some patients might be a safe alternative to SAVR + CABG, at least in patients with non-complex CAD and intermediate surgical risk, as has been recently demonstrated in a randomized study by Sondergaard and colleagues [64]. Additionally, a propensity-matched substudy from the Observant registry (mean Euroscore II 7.8%) found that TAVI + (a priori or concomitant) PCI is comparable to SAVR + CABG with respect to all-cause mortality, myocardial infarction, stroke and unplanned hospitalization at 3-year follow-up [65]. Medical management of CAD in the setting of severe AS is also a possibility if the patient is stable and asymptomatic, especially as there are limited and somewhat contradictory data with regard to the prognostic value of CAD in patients undergoing TAVI; the procedural risk of PCI in the presence of severe AS, and the timing of PCI.

Nevertheless, some medications should also be used with caution, as some of them, such as antianginal drugs, may have adverse effects on the hemodynamic status in AS patients.

## 6. Prognostic Importance of CAD in Patients Undergoing TAVI

The prognostic value of CAD in patients undergoing TAVI is controversial. As the TAVI procedure is increasingly used in low-risk AS patients this issue becomes increasingly important. Type of presentation of existing CAD (stable patient or patient with acute coronary syndrome), clinical evidence, severity of CAD (simple lesion, calcified lesion, chronic total occlusion...) and extent of myocardial ischemia should be taken into account when deciding about potential PCI in AS setting [66].

So far, several studies have addressed the issue of prognostic importance of CAD in patients undergoing TAVI. Stefanini et al. showed that the higher the Syntax score the worse the prognosis 1-year after TAVI [66]. Patients with a Syntax score >22 were particularly at risk of future adverse event. Furthermore, patients with a high Syntax score received less complete revascularization, while patients within the highest tertile (>14) of residual Syntax score were associated with higher rates of cardiovascular death, stroke or myocardial infarction [67]. The WIN-TAVI study showed that in women undergoing TAVI, CAD, either treated with PCI or not, was associated with poor long-term outcomes [68]. Similar findings were observed in a study by Koskinas et al. [69], in which TAVI was associated with periprocedural myocardial injury according to VARC-2 criteria [70], and this was associated with increased 30-day and 2-year mortality. Rodes-Cabau et al. evaluated the incidence and prognostic value of myocardial injury after TAVI, and showed that elevated CK-MB and troponin levels post-TAVI were associated with less improvement in left ventricular ejection fraction (LVEF) and higher cardiac mortality at follow-up, though CAD “per se” was not a predictor of elevated cardiac enzymes post-TAVR [71]. In the SOURCE XT registry [72], the presence of CAD at baseline was associated with a strong trend towards higher 1-year mortality after Edwards SAPIEN XT valve implantation, (HR 1.22; 95% CI (1.00–1.49), *p* = 0.0552). In line with this, researchers from the Bern University Hospital analyzed three age- and gender- matched cohorts of 248 subjects each, and found a significantly increased rate of major adverse cardiovascular and cerebrovascular events (MACCE) at one year in patients undergoing TAVI with concomitant CAD (16.8%) compared with TAVI without CAD (9.8%), and stable CAD undergoing PCI without AS (9.5%), primarily due to a higher risk of cardiovascular death [6]. Results from the German TAVI registry demonstrated a higher rate of in-hospital mortality and lower unadjusted 30-day survival in AS patients with CAD compared with those without CAD. However, this difference was no longer significant after adjusting for confounding factors [73]. A meta-analysis by Witberg et al. including six observational studies and 3110 patients has shown a significant association between non/incomplete revascularization and mortality [74]. Their results revealed that in patients with multivessel disease selective revascularization prior to TAVI with residual Syntax score <8 leads to a better outcome compared to incomplete revascularization with residual Syntax score >8. The mortality risk in the former group was equivalent to those who were without CAD.

Conversely, in a study by Van Mieghem et al., revascularization status did not affect 1-year survival in patients undergoing TAVI, although it should be noted that average Syntax score of included patients was only 9 [75]. Aktug et al. found that 30 day mortality for patients undergoing TAVI plus PCI was similar to patients with isolated TAVI (12.1% vs. 9.9%; OR = 1.4, 95% CI 0.6–3.267; *p* = 0.436) [76]. Results from the UK TAVI registry outlined that CAD, albeit associated with greater comorbid conditions, predicted neither short- nor long-term survival. Similarly, in the ADVANCE study, neither CAD, nor history of myocardial infarction or prior revascularisation were found to be predictors of mortality at 12 months post TAVI [77]. A recent meta-analysis including nine studies and 3858 patients demonstrated no benefit of concomitant or a priori PCI with regard to cardiovascular death, myocardial infarction and acute kidney injury in patients undergoing TAVI. Another meta-analysis, from D’Ascenzo et al., encompassing >8300 patients investigated the impact of extent of CAD and PCI on 30-day and one-year mortality after TAVI [78]. Results demonstrated that significant CAD did not impact on all-cause death after one month or one-year follow-up, yet severity of CAD, assessed by Syntax score >22 was associated with mortality.

In large, there are contradictory data with regard to the prognostic importance of CAD in patients undergoing TAVI, although the majority of studies have demonstrated the prognostic importance of obstructive CAD in the setting of severe AS. Patients with severe AS and concomitant CAD are often complex due to the heterogeneous CAD disease, frequent association with various comorbidities, but also due to the difference in severity and extent of myocardial ischemia, which may explain the different results between the studies. For this purpose, until functional evaluation of the lesion and ischemia is properly validated in AS patients, stratifying patients according to CAD disease severity (i.e., by means of Syntax score and clinical presentation) may allow more accurate assessment of the prognostic implications of CAD on clinical outcomes in TAVI candidates. It should be also noted that none of the above cited studies were randomized, with different patient risk-stratification and without long-term follow-up (>5 years), hence limiting the definite conclusion with regard to prognostic importance of CAD in patients undergoing TAVI.

## 7. PCI Timing in Stabile Patients with AS

In AS patients at high risk of morbidity and mortality from SAVR or those with temporary contraindications to SAVR, such as acute coronary syndromes, or when symptoms are felt to be mainly from CAD, PCI improves survival and quality of life. Yet, there is a paucity of data regarding the best elective PCI timing in stabile patients planned for TAVI. PCI could be performed before, concomitantly with TAVI, or after the TAVI procedure, and for all three options pros and cons can be found in Table 1.

In the study by Gasparetto et al. 113/191 patients undergoing TAVI had CAD, and PCI was performed prior to TAVI in 39 patients [79]. Patients with chronic total occlusions and lesions in vessels <2.5 mm were excluded. They found that 30-day mortality was similar between patients treated with PCI prior to TAVI and patients without CAD (5.7% vs. 2.9%, *p* = ns). The safety of PCI in untreated AS has also been demonstrated in a study by Goel et al. [80]. They retrospectively compared short-term PCI outcome in 254 patients with severe AS and 508 propensity matched patients without severe AS who underwent PCI during the same period. PCI was performed both in the setting of acute coronary syndrome and as an elective procedure. Thirty-day mortality was similar between groups (4.3 vs. 4.7%, HR 0.93, 95% CI 0.51–1.69, *p* = ns), however, a sub-analysis revealed that patients with severe AS, with STS score >10 and LV ejection fraction <30%, had significantly higher 30-day mortality (15.4% and 10.4%, respectively, *p* < 0.05). The main causes of death in the STS score high-risk group were heart failure and multisystem failure. The safety of unplanned PCI in both acute and chronic coronary event has been further accentuated in the recently published international registry [81]. PCI success was reported in 96.6% of patients with no significant differences between patients treated with balloon-expandable and self-expandable bioprostheses (100% vs. 94.9%; *p* = 0.150). Furthermore, an observational retrospective study by Ochiai et al. failed to demonstrate any significant difference in MACCE at two years according to PCI timing in AS patients undergoing TAVI [82]. Of note, the vast majority of patients were treated with balloon-expandable valves, which certainly imposes a limitation on results applicability. Similarly, in the US based registry in which 380 patients were treated with TAVI and PCI, the timing of PCI—either before or concomitant/after TAVI—had no impact on MACCE-free survival, and a propensity-matched analysis of 159 patients yielded similar results [83,84]. Yet, there was a significant discrepancy in terms of timing and number of patients, with 327 patients treated with PCI in the year before TAVI, 38 patients treated with concomitant TAVI and PCI and only 15 patients underwent PCI within two months post TAVI. Finally, the results from ACTIVATION trial (Percutaneous coronary intervention prior to transcatheter aortic valve implantation: a randomized controlled trial, ISRCTN75836930) were recently presented [85]. This is the first randomized study evaluating the importance of PCI in patients with CAD who are planned for TAVI and authors have found no differences with regard to the primary endpoint of death and rehospitalization at one year in 119 patients with severe AS who underwent PCI prior to TAVI vs. 116 who underwent only TAVI (41.5% in the PCI group vs. 44% in non-PCI group, *p* = 0.067). In addition, authors reported a higher rate of bleeding in the PCI group (44.5% vs. 28.4%, *p* = 0.02). Several studies, however, documented difficult coronary cannulation post-TAVI, and its relation to the type of implanted prosthesis [86,87,88]. A recent study assessed post-TAVI coronary access with MDCT coronarography in 66 patients treated with Evolut R or Evolut PRO valves and 345 patients treated with SAPIEN 3 valves [87]. The distance from inflow of the implanted prosthesis to the coronary ostia and the overlap between valve commissures and the coronary ostia were analyzed. Coronary access was defined as unfavorable if the coronary ostium was below the skirt or in front of the prosthesis commissural posts above the skirt. MDCT identified unfavorable coronary access more often in patients with self-expandable valves (34.8% for the left coronary artery and 25.8% for the right coronary artery) compared to balloon-expandable valves (15.7% for the left coronary artery and 8.1% for the right coronary artery). In both groups the success rates of selective coronary cannulation of both arteries were significantly lower in patients with MDCT-diagnosed unfavorable coronary access compared with those with favorable coronary access (Evolut R/Evolut PRO, 0.0% vs. 77.8%, *p* = 0.003; SAPIEN 3, 33.3% vs. 91.4%, *p* = 0.003). This is not to be neglected, as difficulties in coronary arteries cannulation complicates the procedure in terms of fluoroscopy duration and amount of contrast used, as well as possible amount of material used for the procedure. Furthermore, in patients with acute myocardial infarction, time to coronary artery opening is of the essence. On the other hand, in stable patients, functional assessment of stenosis significance is more reliable after TAVI than in patients with untreated AS [48]. Ongoing “Optimal Timing of Transcatheter Aortic Valve Implantation and Percutaneous Coronary Intervention”—The TAVI PCI Trial should shed additional light on optimal PCI timing in patients undergoing TAVI. In this trial (NCT04310046) patients with severe AS and concomitant CAD accepted for TAVI and PCI by the Heart Team will be randomized in a 1:1 ratio to either FFR-guided complete coronary revascularization before (within 1–40 days) or after (within 1–40 days) TAVI using the Edwards SAPIEN Transcatheter Heart Valve^®^ (Edwards Lifesciences Corporation, Irvine, CA, USA).

At this point, although available data are severely limited and only one randomized trial has been presented, it seems that if PCI is the preferred mode of revascularization, when feasible, PCI prior or concomitant with TAVI might be the most logical choice. The Interventional section leadership council of the American College of Cardiology proposed that PCI should be performed in patients with proximal epicardial or left main stenosis if PCI risk is not prohibitive, or in non-proximal stenosis if there is a concern that the patient’s symptoms are caused by CAD [66]. In both cases they recommend PCI prior or concomitant with TAVI. Left main and right coronary artery ostial lesion require special consideration in TAVI candidates because the implanted valve can crush the stent frame. Although technically feasible to perform PCI after TAVI, as already cited, the cannulation of coronary artery and manipulation and delivery of stents may be more challenging. Eventually, each additional PCI procedure adds further risk of adverse events, particularly in the presence of heavily calcified disease and in patients with chronic kidney disease [87].

In patients who are considered for PCI with hemodynamically unstable AS, balloon aortic valvuloplasty may be considered to stabilize the patient before PCI. Actually, balloon aortic valvuloplasty is mostly used nowadays as a bridge to final therapy in unstable patients or patients who cannot be treated with TAVI or SAVR, regardless of the presence of associated CAD.

Hybrid SAVR/PCI procedure is also a treatment possibility. This strategy divides the high-risk surgery into two potentially lower-risk procedures, though the need for dual antiplatelet therapy following PCI would delay the timing of SAVR. A relatively small study which included 123 patients investigated a combining approach of PCI and minimally invasive SAVR demonstrated excellent safety and mid- and long-term outcome [89]. Yet, the applicability of these results should be interpreted with caution as it was a small study performed in high-volume center. Of note, a single-center retrospective study by Byrne et al. [90] was performed in 26 patients who underwent PCI for ACS (24 patients) or for a complex re-operative valve surgery (two patients) followed by aortic/mitral valve surgery using either a minimally invasive or traditional sternotomy approach. Valve replacement was performed a median of five days after PCI, and operative mortality was 3.8%, significantly lower than the STS-predicted mortality of 22%. Of note, there was a high rate of blood transfusion (85%), likely due to the use of dual anti-platelet therapy following PCI. While this study was limited by its small sample size and heterogeneous patient population, it showed the feasibility of performing PCI prior to SAVR. On the other hand, the results of Witberg et al. meta-analysis [73] somewhat diminished the need to perform hybrid TAVI/CABG approach because the combination of reasonable PCI (if complete revascularization is not needed or possible) + TAVI is more convenient and probably preferable to patients.

**Figure 2:**
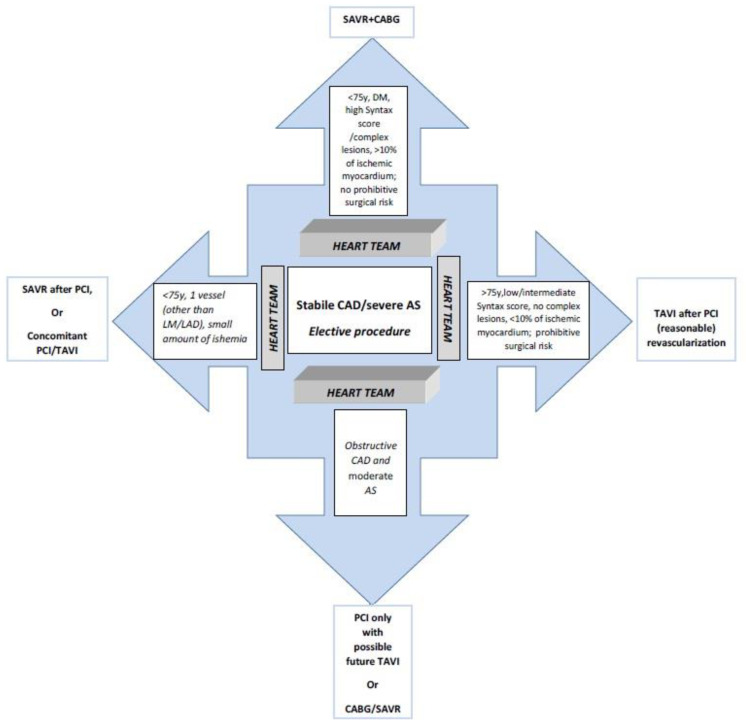
Proposed treatment algorhythm for patients with concomitant coronary artery disease (CAD) and severe aortic stenosis (AS). Of note, recently published 2020 ACC/AHA guidelines state that TAVI can be considered from 65 years of age in patients with no anatomic contraindications for transfemoral TAVI and after shared decision-making about the balance between expected patient longevity and valve durability [92]. SAVR: Surgical aortic valve replacement; CABG: Coronary artery bypass grafting; DM: Diabetes mellitus; LM: left main coronary; LAD: left anterior descending artery; CAD: Coronary artery disease; AS: Aortic stenosis; TAVI: Transcatheter aortic valve implantation; PCI: Percutaneous coronary intervention.

In conclusion, there are different options when treating patients with concomitant AS and CAD. Hereby we propose an algorithm for the treatment approach to these complex patients in Figure 2. However, given that there is no reliable scientific evidence regarding the best approach to the elective treatment of CAD/AS patients, it is necessary to establish a Heart team for decision making. As pointed out, its role in evaluating the candidate and choosing the method and time of treatment is indispensable [91,92].

## Figures and Tables

**Table 1:** PCI timing in TAVI candidates: pro and cons.

	Pros	Cons
PCI before TAVI	Possible alleviation of symptoms in elderly and avoidance of TAVI procedure Easier access to coronary arteries	Acute decompensation Left main and right coronary artery ostial lesion require special consideration because an implanted valve can crush the stent frame.
PCI concomitant with TAVI	If procedures are straightforward it is most comfortable for the patient Reduction of vascular bleeding Lower costs	Higher amount of contrast Higher radiation dose
PCI after TAVI	More accurate assessment of the functional severity of CAD	Cannulation of coronary artery and manipulation and delivery of stents may be more challenging Calcified lesions requiring rotational atherectomy may be very challenging (possible interaction between the rota system and TAVI frame) Longer-term dual antiplatelet therapy may be applied more safely

PCI, percutaneous coronary intervention, TAVI, transcatheter aortic valve implantation.

## Data Availability

Not applicable.
